# Detection of Influenza A(H3N2) Virus RNA in Donated Blood

**DOI:** 10.3201/eid2607.200549

**Published:** 2020-07

**Authors:** Rafael dos Santos Bezerra, Daniel Macedo de Melo Jorge, Ítalo Araújo Castro, Edson Lara Moretto, Leonardo Scalon de Oliveira, Eugênia Maria Amorim Ubiali, Dimas Tadeu Covas, Eurico Arruda, Simone Kashima, Svetoslav Nanev Slavov

**Affiliations:** Blood Center of Ribeirao Preto, Ribeirao Preto, Brazil (R.S. Bezerra, E.L. Moretto, L.S. Oliveira, E.M.A. Ubiali, D.T. Covas, S. Kashima, S.N. Slavov);; Faculty of Medicine of Ribeirao Preto–University of São Paulo, Ribeirao Preto (D.M.M. Jorge, I.A. Castro, E. Arruda)

**Keywords:** Influenza A, H3N2, RNA, viral metagenomics, blood donations, viruses, influenza, respiratory infections

## Abstract

Influenza A virus infection has rarely been documented to cause viremia. In 28 blood donations in Brazil that were deferred because of postdonation information, we identified influenza A(H3N2) virus RNA in 1 donation using metagenomic analysis. Our finding implies theoretical risk for viremia and transfusion transmission.

Influenza A virus is easily transmitted by respiratory aerosols and droplets and has high epidemic and pandemic potential (*1*). Influenza viremia may be established within 2–3 days before onset of clinical symptoms (*2*), implying that this virus is hypothetically transmissible by blood transfusion. However, transmission has never been confirmed, and efforts to detect influenza virus RNA among blood donors have been unsuccessful (*3*–*5*).

We performed next-generation sequencing and viral metagenomic survey in plasma samples aiming to identify viruses potentially associated with adverse effects reported by blood donors up to 14 days after donation (fever, exanthema, headache, myalgia, diarrhea, jaundice, sore throat, cough, conjunctivitis), which is defined as postdonation information. Such information generally results in disposal of the donated blood to prevent transfusion transmission of not routinely tested infectious agents.

During 2019, we performed Illumina sequencing (https://www.illumina.com) on blood donations identified through postdonation information in the Ribeirao Preto Blood Center (Ribeirao Preto, Brazil). We manually extracted viral nucleic acids from plasma using the High Pure Viral Nucleic Acids kit (Roche, https://www.roche.com) and performed reverse transcription using the SuperScript First Strand Synthesis System (ThermoFisher Scientific, https://www.thermofisher.com) following the manufacturer’s guidelines. We generated cDNA libraries using Nextera DNA Flex Library preparation kit (Illumina) and sequenced them in Illumina NextSeq 550 equipment. We conducted viral metagenomic analysis using a bioinformatic pipeline focused on viral discovery comprising FastQC version 0.11.8 (https://www.bioinformatics.babraham.ac.uk/projects/fastqc), Trimmomatic version 0.3.9 (http://www.usadellab.org/cms/?page=trimmomatic), AfterQC version 0.9.7 (https://github.com/OpenGene/AfterQC), Kraken2 2.0.8 (https://ccb.jhu.edu/software/kraken2), SPAdes version 3.13.0 (http://cab.spbu.ru/software/spades), and Diamond 0.9.29 (https://github.com/bbuchfink/diamond) software.

In 1 (4%) of 28 blood donations, we identified RNA fragments of influenza A. The donor, a 25-year-old woman, donated blood on January 28, 2019, after an interview in which she was approved as eligible for blood donation. Three days later (January 30) she reported low-grade fever, cough, and coryza to the blood center. The blood donation positive for influenza A was processed into packed red cells, plasma, and platelets, but because of the timely postdonation information report, all hemoderivatives were discarded. Because of the low severity of the reported symptoms, which were not directly related to the blood donation procedure, the donor was not followed up.

The viral metagenomic pipeline identified a 756-bp fragment of polymerase base (PB) 2 gene of influenza A, but targeted assembly using Burrow-Wheeler Aligner version 0.6 (http://bio-bwa.sourceforge.net) detected other genomic regions like matrix 1 and 2 (252 bp), nucleoprotein (257 bp), and polymerase acidic frameshift protein (707 bp). We directly confirmed influenza A using multisegment reverse transcription PCR (RT-PCR) as described previously (*6*). We then amplified the hemagglutinin and neuraminidase segments individually by conventional PCR (*7*) using the multisegment RT-PCR product as input volume and sequenced. Consensus sequences of hemagglutinin (477 bp) and neuraminidase (647 bp) were aligned with MAFFT 7.0 software (ThermoFisher Scientific) using a dataset obtained from GISAID (https://www.gisaid.org), selecting strains from Brazil that circulated during 2011–2019. The phylogenetic analysis with IQ-TREE 2.0 software (http://www.iqtree.org) using hemagglutinin (264 sequences) and neuraminidase (419 sequences) applying the K3Pu+F+G4 and TVMe+G4 substitution models demonstrated that the strain clustered with isolates circulating during the 2019 epidemic season. The influenza A in the donated blood was phylogenetically determined to belong to H3N2 type (Figure). We deposited the hemagglutinin sequence in GenBank under accession no. MT126243 and the neuraminidase sequence under accession no. MT126244.

**Figure:**
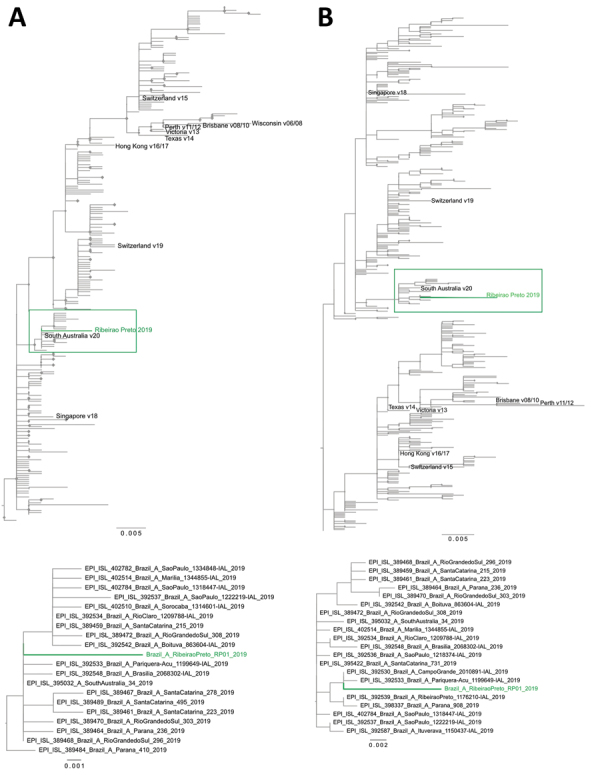
Maximum-likelihood phylogenetic tree of hemagglutinin (A) and neuraminidase (B) of influenza A(H3N2) virus detected in blood donation, Brazil. A total of 264 hemagglutinin and 419 neuraminidase sequences from seasonal strains circulating during 2011–2019 and available in GISAID (https://www.gisaid.org) were used to estimate the phylogenetic relationships with the influenza A virus detected in Ribeirao Preto, Brazil. Green indicates the H3N2 strain obtained from blood donor from Ribeirao Preto; diamonds at each node indicate statistical support along branches defined as ultrafast bootstrapping >90% (of 10,000 replicates). The cluster where the donor strain obtained from Ribeirao Preto is located is shown in detail at bottom. Scale bars indicate nucleotide substitutions per site.

Influenza A viremia has been reported during acute-phase illness, mainly in patients infected with more pathogenic influenza viruses, such as H5N1 (*8*). Our study demonstrates the asymptomatic presence of influenza A virus RNA in blood donors preceding symptom onset, which provides theoretical grounds for the possibility of influenza transmission by blood transfusion. A study performed by the American Red Cross on 1,004 samples from blood donors reporting postdonation influenza symptoms detected no influenza-positive samples by RT-PCR during the first H1N1 outbreak in 2009 in the United States (*5*). Other studies conducted during the 2009 H1N1 pandemic also detected no viral RNA in persons who donated blood during the incubation period and in whom influenza symptoms developed 2−7 days after donation (*3*,*4*).

Although our report is based on a single blood donation, the finding raises the theoretical possibility of transmission of influenza by blood transfusion, which may be of great concern for transfusion services during seasonal influenza outbreaks or pandemics. Unfortunately, we could not confirm whether the detected influenza virus H3N2 remained infectious in the donated plasma. Nevertheless, identification of influenza RNA is especially important because a substantial proportion of persons who receive blood transfusions may have permanent or transient immune dysfunction, which might lead to unfavorable clinical outcome if transfusion-acquired influenza infection occurs. The transmission of influenza by transfusion remains understudied, and more detailed surveys are needed, including assessment of influenza viremia in blood donors during seasonal outbreaks and confirmation of cell-associated peripheral blood circulation of influenza subtypes.
